# Molecular and serological evidence of Nairobi sheep disease virus in *Haemaphysalis longicornis* and sheep in eastern Shandong Peninsula, China

**DOI:** 10.1093/jvimsj/aalag055

**Published:** 2026-03-30

**Authors:** Yingxin Tu, Yujing Tang, Xiaolin Han, Jing Feng, Xinbei Li, Meixi Ren, Jian Song, Xiuwei Feng, Guoyu Niu, Hengyi Sun, Yanyan Wang

**Affiliations:** School of Public Health and School of Life Sciences and Technology, Shandong Second Medical University, Weifang 261053, China; School of Public Health and School of Life Sciences and Technology, Shandong Second Medical University, Weifang 261053, China; School of Public Health and School of Life Sciences and Technology, Shandong Second Medical University, Weifang 261053, China; Weifang People’s Hospital, Shandong Second Medical University, Weifang 261000, China; School of Public Health and School of Life Sciences and Technology, Shandong Second Medical University, Weifang 261053, China; School of Public Health and School of Life Sciences and Technology, Shandong Second Medical University, Weifang 261053, China; School of Public Health and School of Life Sciences and Technology, Shandong Second Medical University, Weifang 261053, China; School of Public Health and School of Life Sciences and Technology, Shandong Second Medical University, Weifang 261053, China; School of Public Health and School of Life Sciences and Technology, Shandong Second Medical University, Weifang 261053, China; School of Public Health and School of Life Sciences and Technology, Shandong Second Medical University, Weifang 261053, China; Department of Clinical Laboratory, Suqian First Hospital, Suqian 223812, China

**Keywords:** phylogenetic analysis, polyclonal antibodies, seroprevalence, tick-borne viruses

## Abstract

**Background:**

Nairobi sheep disease virus (NSDV), a highly pathogenic tick-borne orthonairovirus affecting ruminants, represents an emerging zoonotic threat. While endemic to East Africa, recent reports confirm its presence in Asia.

**Hypothesis/Objectives:**

This study investigated NSDV circulation in the eastern Shandong Peninsula.

**Animals:**

A total of 745 *Haemaphysalis longicornis* ticks (grouped into 139 pools) and 246 ruminant serum samples were collected during 2021 field surveillance in Weifang and Yantai regions.

**Methods:**

Molecular detection employed quantitative reverse transcription PCR and nested PCR assays, with genomic characterization through complete and partial segment sequencing. Serological analysis used ELISA and immunofluorescence assays (IFAs). Phylogenetic reconstruction was performed using maximum-likelihood methods.

**Results:**

Nairobi sheep disease virus RNA was detected in 9 tick pools (minimum infection rate: 1.2%). Genomic analysis revealed one complete genome (L: 11 999 bp; M: 5013 bp; and S: 1469 bp) and 4 partial S segment sequences (975 bp) showing > 98% nucleotide identity with Chinese reference strains and 100% identity among themselves. Phylogenetically, Shandong isolates clustered with other Chinese NSDV variants, indicating local evolutionary adaptation. Seroprevalence reached 5.7% (14/246) among ruminants, suggesting local virus exposure.

**Conclusions and clinical importance:**

This molecular and serological evidence of NSDV in eastern Shandong suggests the potential presence of the virus beyond its historical range. The close genetic relationship with other Chinese strains suggests regional spread rather than independent introductions. These findings underscore the need for enhanced surveillance in tick-ruminant systems and the development of control strategies to mitigate economic and zoonotic risks.

## Introduction

Nairobi sheep disease (NSD) is a significant zoonotic disease caused by the Nairobi sheep disease virus (NSDV), a single-stranded, negative-sense RNA virus belonging to the genus *Orthonairovirus* within the family *Nairoviridae.*^1^ Nairobi sheep disease virus primarily infects sheep and goats, causing severe clinical manifestations including dyspnea, mucopurulent nasal discharge, and hemorrhagic gastroenteritis, with death rates ranging from 30% to 70% in endemic cases and reaching up to 90% during severe outbreaks.^2^ Due to its substantial economic effect by adding to the cost of livestock production, the World Organisation for Animal Health has designated NSD as a notifiable disease.^3^ Initially discovered in Kenya in 1910, NSDV traditionally exhibited endemic distribution across East Africa.^4-7^ However, emerging evidence has revealed viral circulation in Asia, with Ganjam virus confirmed as an Asian genetic variant of NSDV.^8^ China’s first autochthonous NSDV strain was isolated from northeastern provinces in 2013,^9^ with subsequent molecular detection in Hubei and Shandong provinces.^10^^,^^11^

Despite the expanding distribution of NSDV in China, current studies primarily focus on viral nucleic acid detection, with limited seroepidemiological data in animal populations. This knowledge gap constrains our understanding of transmission dynamics and epidemiological patterns in endemic regions. Shandong Province, characterized by extensive cattle and sheep farming operations, lacks systematic NSDV surveillance data, limiting effective disease prevention and control strategies.

This study aimed to investigate NSDV distribution in the eastern Shandong Peninsula (specifically Yantai and Weifang regions) through molecular epidemiological surveillance and serological analysis. We hypothesized that NSDV circulates in local tick populations and elicits seropositive responses in regional animal populations. Our findings will expand existing knowledge on NSDV geographic distribution and provide novel insights into viral epidemiological patterns and transmission dynamics in this economically important agricultural region.

## Materials and methods

### Tick collection and processing

**Figure 1:**
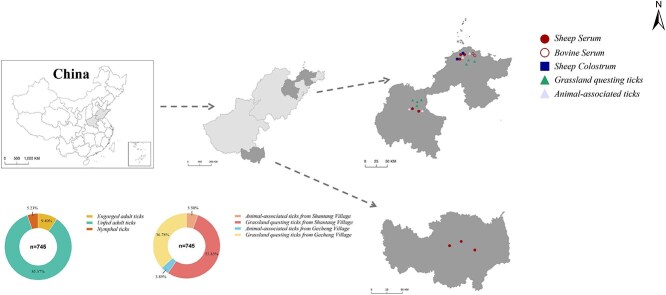
Map of where the tick and animal samples in this study were collected in Weifang and Yantai city, Shandong Province.

From July to September 2021, we collected tick specimens from both the sheep and the surrounding grasslands in Shantang Village (Weifang City) and Ge Cheng Village (Yantai City), Shandong Province (Figure 1). Engorged ticks were carefully removed from domestic animals using sterile forceps, placed in perforated tubes containing moistened filter paper, temporarily stored in cool, ventilated conditions for one week, and subsequently preserved in liquid nitrogen. Questing ticks from grasslands were collected using the standard 100 cm × 100 cm flannel cloth dragging method and immediately frozen in liquid nitrogen. After morphological identification by expert taxonomists, we performed next-generation sequencing (NGS) to analyze mitochondrial cytochrome c oxidase I (*COI*) gene sequences for precise species determination of *Haemaphysalis* ticks. Sequencing reads were aligned against a reference database comprising *COI* genes from 30 *Haemaphysalis* species using Bowtie2 (v2.4.1). For processing, blood-fed ticks were individually homogenized, while unfed specimens were pooled (5-15 ticks per tube) based on collection site, species, and developmental stage.

### Animal serum and colostrum collection

Blood samples (about 5 mL/animal) were collected aseptically from the jugular vein using random sampling and stored in anticoagulant tubes. After centrifugation at 1000 × *g* for 5 min, serum supernatants were separated and stored at 4°C for subsequent analysis. In addition, colostrum was obtained from postpartum ewes within one week of parturition. After strict udder disinfection, 10-20 mL of colostrum was collected aseptically, transported on ice, and centrifuged at 3000 × *g* for 15 min within 4 h to isolate whey. The whey samples were aliquoted and stored at −80°C in an ultra-low-temperature freezer until further testing.

### Nucleic acid extraction

Before extraction, ticks were washed 3 times with phosphate-buffered saline (PBS) to remove surface contaminants. Each sample was homogenized in 800 μL of pre-cooled RPMI-1640 medium using a TissueLyser II homogenizer (Qiagen, Germany) at 40 times for 15 min. The resulting homogenate was centrifuged at 10 000 × *g* for 10 min at 4°C (Eppendorf, Germany), and 160 μL of supernatant was collected for RNA extraction using the TIANamp Virus RNA Kit (Tiangen, China) following the manufacturer’s protocol. For blood and colostrum samples, nucleic acids were extracted directly using the same kit. The purified RNA was either processed immediately or stored at −80°C for subsequent analysis.

### PCR detection of NSDV in ticks

Detection of NSDV was performed using quantitative reverse transcription PCR (qRT-PCR) targeting a conserved region of the viral nucleoprotein gene. The complete S-segment genome sequence of NSDV (retrieved from GenBank) was aligned using Clustal W (BioEdit v7.0.9) to identify highly conserved regions for primer and probe design. Specific primers and probes were designed using Primer Express v3.0 (Applied Biosystems, USA) and validated by BLAST analysis to ensure specificity. All qRT-PCR-positive samples were further confirmed via nested PCR to obtain sequence data. Amplification reactions were conducted in a 20 μL total volume containing 2 μL of template RNA, 0.4 μL of each primer (10 μM), and the RT-PCR master mix. The thermal cycling was performed on a real-time PCR system using the following one-step protocol: reverse transcription at 42°C for 5 min, initial denaturation at 95°C for 10 s, followed by 40 cycles of denaturation at 95°C for 5 s and annealing/extension at 60°C for 30 s,^12^ with primer and probe sequences listed in Table 1. The resulting amplicons were purified by 1% agarose gel electrophoresis and subsequently sequenced by a commercial service (Shanghai Biotech).

**Table 1:** Primers and probe used for identification of NSDV in this study.

**Virus**	**Type**	**Primer/probe**	**Sequence (5′ to 3′)**	**Expected size (bp)**
**NSDV**	qRT-PCR	F	GTCCTAACAGCTGGGAGAATGTCT	/
		R	GATGTGGCCTGAACCCTCA	
		P	CCCAGTTGCCAATCCTGACGATGC	
	Nested PCR	Outer-F	GGTCGAGCGTGGTCTGGACT	1099
		Outer-R	CAAATGCTCCGAGGCAACTATGT	
		Inner-F	GTACGGGAAGCTAAGAACGGAAGT	976
		Inner-R	GAATAGGGCGACGATGTTCTGT	

Abbreviations: qRT-PCR = quantitative reverse transcription PCR; NSDV = Nairobi sheep disease virus.

### Genome sequencing and analysis

Viral genomic RNA samples were pooled equally into 3 groups for high-throughput sequencing. Based on RNA concentration, samples were processed differentially: those with > 10 ng/μL underwent ribosomal RNA depletion using the FastSelect rRNA Removal Kit (Novozymes), while lower-concentration samples were processed directly. Sequencing libraries were prepared with the VAHTS Universal V8 RNA-seq Library Prep Kit (Novozymes), followed by quality assessment via Agilent 2100 Bioanalyzer and qPCR (Geneplus-Beijing Clinical Laboratory). Paired-end sequencing (2 × 150 bp) was performed on a DNBSEQ-T7 platform. Raw reads were quality-filtered, assembled using MegaHit (length threshold > 200 bp), and annotated via BLASTx (E-value = 1) against the nr database (December 2024 release). Key NSDV genomic regions were validated by Sanger sequencing using target-specific primers to ensure data accuracy.

### Virus isolation

Tick homogenates positive for NSDV nucleic acid were filtered through a 0.45 μm membrane to remove bacteria and fungi. The clarified supernatant was used as viral inoculum. Susceptible cell lines (BHK-21) were seeded to 80% confluency, after which the medium was aspirated and replaced with 1 mL of inoculum. After adsorption at 37°C for 1 h (with intermittent gentle agitation), fresh medium was added, and cultures were maintained for 14 days. Daily observations were recorded for cytopathic effects (eg, cell rounding, shrinkage, and detachment). To confirm viral propagation, 3 blind passages were performed on all samples.

### Plasmid construction and purification of nucleoprotein

The full-length S segment of NSDV was amplified by PCR using reverse-transcribed cDNA as template, then cloned into pET30a vector (NcoI/XhoI sites) with an N-terminal His-tag. After sequence verification in DH5α competent cells, the recombinant plasmid was transformed into BL21(DE3) cells for protein expression. Induction was performed with 0.5 mM IPTG at 25°C for 6 h. Bacterial cells were harvested and resuspended in binding buffer (20 mM Tris–HCl, 500 mM NaCl, 20 mM imidazole, pH 8.0), followed by ultrasonic lysis (200 W, 3 s pulse/5 s interval, 5 min total duration on ice). After centrifugation (12 000 × *g*, 20 min), the supernatant was loaded onto a Ni-NTA column. The column was washed with Wash Buffer (40 mM imidazole) before eluting the target protein with elution buffer (250 mM imidazole). The purified protein was dialyzed against PBS to remove imidazole, concentrated by ultrafiltration, and verified by sodium dodecyl sulfate polyacrylamide gel electrophoresis (SDS-PAGE) and Western blot analysis.

### Animal immunization and purification of polyclonal antibody

For polyclonal antibody production, 3-month-old New Zealand White rabbits were immunized following a standard protocol. The primary immunization consisted of 0.5 mg purified protein antigen emulsified with complete Freund’s adjuvant, administered via subcutaneous injection at 8-10 dorsal sites (0.1-0.2 mL per site). Three booster immunizations were subsequently performed at 7-14 day intervals using 0.3 mg antigen emulsified with incomplete Freund’s adjuvant, delivered through both subcutaneous (4 dorsal sites) and intramuscular routes (0.5 mL per hind limb). Serum was collected 7 days post-final immunization through either marginal ear vein puncture or carotid artery exsanguination. After centrifugation, the antiserum was aliquoted and stored at −80°C. All procedures were conducted under aseptic conditions with regular monitoring of animal health and immune responses. Antibody titers were monitored throughout the immunization schedule to evaluate the immune response.

The polyclonal antibody purification process followed standardized procedures: Primary purification was achieved through Protein A/G affinity chromatography, leveraging the specific interaction between antibody Fc regions and Protein A/G. Samples in pH 7.4 binding buffer (PBS) were loaded onto the column, followed by elution using 0.1 M glycine-HCl (pH 2.5-3.0) with immediate neutralization in 1 M Tris–HCl (pH 8.5). Secondary purification employed ion-exchange chromatography (DEAE or SP columns) to remove residual heteroproteins. The purification process was monitored by absorbance at 280 nm, with final product purity and specificity confirmed by SDS-PAGE and Western blot analysis. Purified antibodies were sterilized through 0.22 μm filtration and stored at −80°C in 50% glycerol (v/v) for long-term preservation, while aliquots in PBS were maintained at 4°C for short-term use.

### Indirect ELISA for detection of NSDV-NP specific antibodies

The purified NSDV nucleoprotein (NP) was diluted to 2 μg/mL in coating buffer and 100 μL (200 ng/well) was coated onto 96-well polyvinyl chloride microplates (Corning) overnight at 4°C. After blocking with 3% bovine serum albumin (200 μL/well) for 1 h at room temperature, plates were washed 5 times with PBS with Tween 20. Test samples (serum or colostrum) were diluted 1:100 in dilution buffer and incubated (100 μL/well) at 37°C for 30 min. After washing, species-specific horseradish peroxidase-conjugated secondary antibodies (Huaxing Biochuang; 1:2000 dilution) were added and incubated at 37°C for 1 h. After final washes, color development was performed using 3,3′,5,5′-tetramethylbenzidine substrate (50 μL each of solutions A and B) for 5 min at room temperature before stopping the reaction with 100 μL stop solution, and the absorbance value was measured at 450 nm. The cutoff value was calculated as the mean optical density (OD) of negative control sera plus 3 SDs. All experimental procedures were performed in triplicate.

### Validation of ELISA-positive specimens by indirect immunofluorescence assay

The eukaryotic expression plasmid NSDV-NP-pcDNA3.1 was constructed and sequence-verified before transfection. HEK293T cells at 75% confluence were transfected with 6 μg recombinant plasmid using lipofectamine and cultured for 24-48 h at 37°C with 5% CO₂. Cells were harvested, washed with PBS, and resuspended to 1 × 10^6^ cells/mL. Cell suspensions (50 μL) were spotted onto multi-well slides, air-dried, and fixed with ice-cold acetone for 10 min. For immunofluorescence staining, slides were incubated with 1:100 diluted test samples (primary antibodies) at 37°C for 30 min in a humidified chamber. After PBS washes, fluorescein isothiocyanate (FITC)-conjugated secondary antibodies (goat anti-rabbit IgG or rabbit anti-sheep IgG, 1:100 dilution) were applied under identical conditions. After final washes, slides were mounted with glycerol and examined by fluorescence microscopy. Appropriate controls included pcDNA3.1-transfected (negative control) and untransfected cells (blank control). All experiments were performed in triplicate to ensure reproducibility.

### Data analysis

For NSDV prevalence in ticks, we calculated the minimum infection rate (MIR) by dividing the number of positive pools by the total number of tested ticks, expressed as infections per 100 ticks. Regional prevalence differences were analyzed using chi-square tests (GraphPad Prism 9.0) with statistical significance set at *P* < .05. Phylogenetic analysis of NSDV-NP gene fragments was performed using maximum likelihood method (MEGA 11.0) with 1000 bootstrap replicates; nodes with ≥ 75% support were considered robust. Reference sequences from *Nairoviridae* family members were retrieved from GenBank via BLASTN alignment. Sequence similarity and genetic distance analyses were conducted using BioEdit 7.2. The newly identified NSDV sequences have been deposited in GenBank under accession numbers PQ684032-PQ684034 and PV520060-PV520063.

## Results

### Tick collection and NSDV detection

A total of 745 ticks were collected from animal hosts and grasslands in Shantang Village (Weifang) and Ge Cheng Village (Yantai), Shandong Province, comprising 70 animal-parasitizing and 675 free-living specimens (Figure 1; Table 1). Macrotranscriptome sequencing revealed > 99% sequence similarity to *Haemaphysalis longicornis* in all libraries, with morphological identification confirming this species composition (70 blood-engorged adults, 636 unfed adults, and 39 nymphs). The findings demonstrate *H longicornis* dominance in the local tick population. Specimens were categorized into 139 pools based on collection site and feeding status, with 9 pools testing positive for NSDV by qRT-PCR (MIR: 1.2%) (Table 2). All 9 qRT-PCR-positive samples were subjected to nested PCR for sequence confirmation. Among them, 4 samples yielded positive bands and were successfully sequenced. The remaining 5 samples tested negative in nested PCR, likely due to low viral loads (*Ct* values > 32.0 in qRT-PCR) or partial RNA degradation during sample processing, which falls below the detection limit of the conventional nested PCR assay. No significant difference in NSDV positivity was observed between regions (Weifang: 5/442; Yantai: 4/303). All tested animal sera and colostrum samples were NSDV-negative by nucleic acid detection.

**Table 2:** qRT-PCR to determine NSDV in tick samples of Weifang and Yantai, Shandong Province, in 2021.

**Collection site**	**Origin**	**No. of individuals**	***Haemaphysalis longicornis* (engorged adults/unfed adults/nymphs)**	**No. of pools**	**qRT-PCR positive**	**Nested-PCR positive**	**MIR (%)**	***P***
**Weifang**	Animal	442	41/0/0	41	3	3	1.1	>.05
	Grassland		0/369/32	41	2			
**Yantai**	Animal	303	29/0/0	29	2	1	1.3	
	Grassland		0/267/7	28	2			
**Total**		745	70/636/39	139	9	4	1.2	

Abbreviations: MIR, minimum infection rate; qRT-PCR, quantitative reverse transcription PCR; NSDV, Nairobi sheep disease virus.

### Comparative analysis of NSDV genomes

Next-generation sequencing of 3 libraries generated substantial data outputs (8.65, 5.47, and 5.38 Gb) with corresponding read counts of 28.84, 18.22, and 17.92 million, respectively. Comprehensive analysis of library 1 successfully assembled the complete coding sequences of all 3 NSDV genomic segments: (1) The L segment (11 999 bp) contains an open reading frame (ORF; positions 17-11992) encoding the RNA-dependent RNA polymerase (RdRp); (2) the M segment (5013 bp) features an ORF (30-4916) encoding the glycoprotein precursor (GP); and (3) the S segment (1469 bp) includes an ORF (15-1463) encoding the nucleocapsid protein. The sequences have been uploaded to GenBank with the accession numbers PQ684032.1-PQ684034.1.

**Figure 2:**
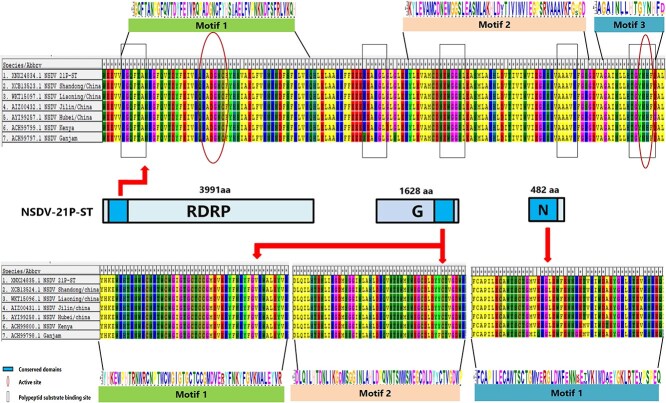
Comparison of important protein binding sites for the conserved structural domains found on the 3 NSDV genome segments. Regions and residues corresponding to viral conserved structural domains are represented in different frames. Abbreviation: NSDV = Nairobi sheep disease virus.

Functional characterization of NSDV-21P-ST revealed conserved genomic features consistent with known isolates. The L segment encodes an RdRp protein exhibiting characteristic Bunyavirus structural domains, including conserved polypeptide substrate binding and active sites. While the glycoprotein (M segment) and nucleoprotein (S segment) lacked currently identified functional binding sites, both demonstrated stable conserved domains. Comparative analysis of 7 geographically distinct NSDV strains showed remarkable conservation in the L segment, with minimal amino acid variation across global sequences (Figure 2), indicating slow evolutionary rates for these core polymerase domains. Notably, the Indian Ganjam strain displayed unique mutations in both M and S segments, suggesting localized adaptive evolution of these viral proteins. These findings highlight differential evolutionary pressures acting on NSDV genomic segments, with structural proteins showing greater plasticity than the highly conserved polymerase.

### Phylogenetic analysis

**Figure 3:**
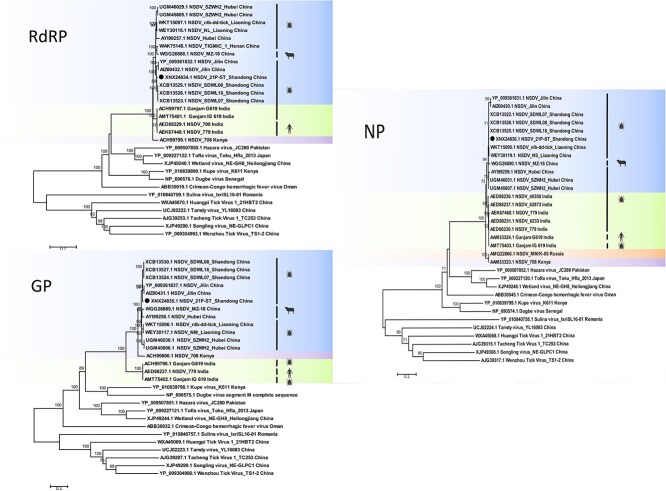
Phylogenetic relationships of NSDV and related *Nairoviridae* viruses were reconstructed using ML methods based on amino acid sequences of the RdRp, GP, and NP. Bootstrap values (>70%) are shown at branch nodes. Sequences obtained in this study are indicated by solid black circles. Abbreviations: GP = glycoprotein; ML = maximum likelihood; NSDV = Nairobi sheep disease virus; NP = nucleocapsid protein; RdRp = RNA-dependent RNA polymerase.

After comparing the NSDV genomes with those reported globally, the nucleotide homology of the NSDV sequences obtained in this study ranged from 94.2% to 98.8% with strains detected in other regions of China, from 87.1% to 87.3% with Kenyan and Russian strains, and a relatively low identity (74.4%-89.3%) with Indian strains. In a phylogenetic tree based on the proteins encoded by the 3 fragments, all reported NSDVs were classified into 3 phylogenetic groups (Figure 3). The first group includes strains from China, the second group includes strains found in India, while the third group includes strains from Kenya and Russia. Notably, while Chinese NSDV strains clustered closely with Indian strains in RdRp and NP phylogenies, their GP protein showed greater similarity to Kenyan/Russian strains, suggesting potential historical recombination events. The 21P-ST-20 strain exhibited closest evolutionary proximity to Shandong and Jilin isolates, indicating a common ancestral origin. In addition, partial S-segment sequencing (975 bp) from 4 qRT-PCR-positive samples demonstrated complete nucleotide identity (100%), further supporting minimal genetic divergence among local circulating strains.

### Purification of recombinant NSDV nucleoprotein and polyclonal antibody

**Figure 4:**
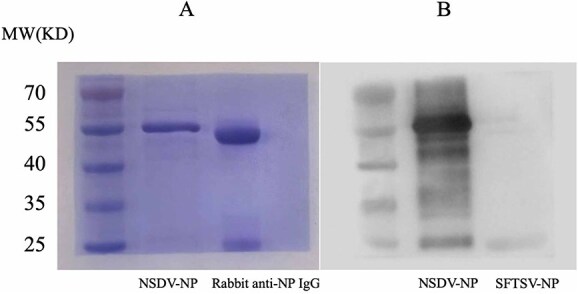
Purification and characterization of NSDV nucleoprotein and related polyclonal antibody. (A) SDS-PAGE analysis. (B) Western blot validation of NSDV and SFTSV nucleoprotein using rabbit polyclonal anti-NSDV-NP antibodies. Abbreviations: NP = nucleocapsid protein; NSDV = Nairobi sheep disease virus; SFTSV = severe fever with thrombocytopenia syndrome virus; SDS-PAGE = sodium dodecyl sulfate polyacrylamide gel electrophoresis.

The NSDV-NP-pET30a recombinant plasmid was successfully expressed in *Escherichia coli* BL21(DE3), yielding predominantly insoluble inclusion bodies. After IPTG induction, the His-tagged fusion protein was purified under denaturing conditions (8 M urea) using Ni-NTA affinity chromatography. Protein renaturation was achieved through stepwise dialysis against PBS buffers containing progressively decreasing urea concentrations (6 M → 4 M → 2 M → urea-free). Subsequent ultrafiltration concentration yielded 34.8 mg of soluble protein per liter of bacterial culture. Sodium dodecyl sulfate-PAGE analysis confirmed high purity (>90%) of the recombinant protein, exhibiting a single band at the expected molecular weight (Figure 4A). This purified antigen was subsequently used for polyclonal antibody production.

New Zealand White rabbits immunized with purified NSDV-NP antigen exhibited transient local swelling at injection sites that resolved within 48-72 h, with no systemic adverse effects observed. Terminal bleeds performed 7 days post-final immunization yielded 10 mL of antiserum, from which approximately 20 mg of IgG was purified via Protein A/G affinity chromatography. Sodium dodecyl sulfate polyacrylamide gel electrophoresis analysis confirmed antibody purity > 90% (Figure 4A). Western blot validation demonstrated specific recognition of NSDV-NP without cross-reactivity to nontarget proteins (Figure 4B), confirming the antibody’s high specificity for diagnostic applications.

### Indirect ELISA for the detection of antibodies in animal specimens

**Figure 5:**
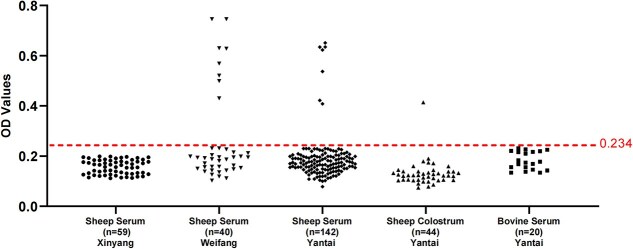
Indirect-ELISA method based on NSDV nucleoprotein. Definition of the cut-off value through tests on 59 serum samples from Xinyang. The dashed horizontal line indicates the cut-off value of the assay. Two hundred forty-six animal samples from different regions were tested using this method. Abbreviation: NSDV = Nairobi sheep disease virus.

We developed an indirect ELISA assay utilizing prokaryotically expressed NSDV-NP protein to assess local animal exposures. The diagnostic cutoff value (0.23) was established using 59 confirmed negative sheep sera from Xinyang (Henan Province), calculated as mean OD + 3 SDs (Figure 5). Among 246 tested samples (diluted 1:100), 16 showed seropositivity (6.5%). Significant regional variation was observed, with Weifang sheep sera demonstrating higher seroprevalence (20%, 8/40) compared to Yantai samples (4.9%, 7/142; *P* < .05). Low-level positivity was detected in Yantai sheep colostrum (2.3%, 1/44), which was collected from a flock where seropositive adult sheep were also identified, while all cattle sera (*n* = 20) were negative. These findings, validated using rabbit anti-NSDV-NP polyclonal antibody as positive controls, provide crucial epidemiological insights into NSDV circulation patterns in Shandong Province.

### Validation of ELISA-positive samples by indirect immunofluorescence assay

**Figure 6:**
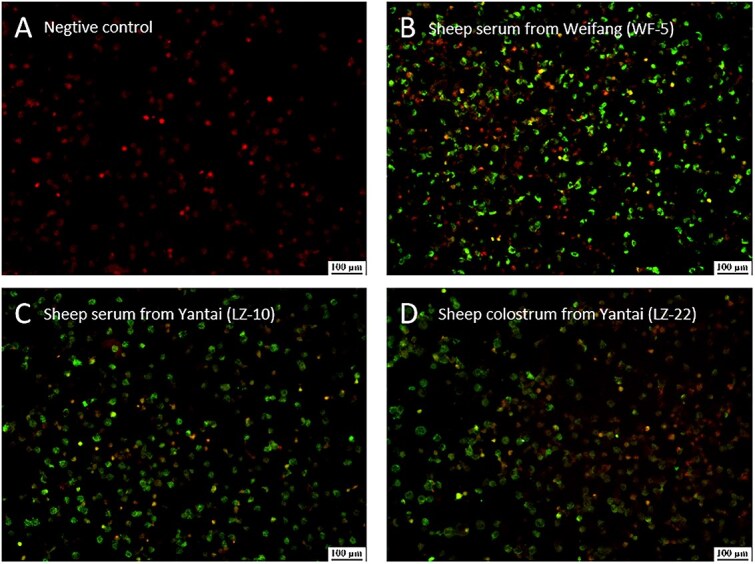
Indirect IFA was used to validate 16 animal samples that tested positive for ELISA. (A) A representative sheep serum from Xinyang was used as a negative control. (B) A representative of sheep serum from Weifang. (C) A representative of sheep serum from Yantai. (D) Sheep colostrum from Yantai. Abbreviation: IFA = immunofluorescence assay.

To confirm ELISA results, we performed immunofluorescence assay (IFA) testing on all 16 ELISA-positive samples (8 from Weifang, 7 from Yantai, 1 colostrum) and 10 negative controls. Antigen slides were prepared using NSDV-NP-transfected HEK293T cells, with serum samples (1:100 dilution) detected using FITC-conjugated secondary antibody (1:1000). Immunofluorescence assay confirmed 87.5% (14/16) of the ELISA positive samples, showing characteristic cytoplasmic fluorescence that strongly correlated with ELISA OD450 values (*r* = 0.82, *P* < .01) (Figure 6). Regional differences emerged, with Weifang samples exhibiting significantly higher fluorescence intensity (125.6 ± 18.3) versus Yantai (89.4 ± 12.7; *P* < .05). Method comparison revealed 87.5% agreement (κ = 0.81), with IFA demonstrating 100% specificity (10/10) and 87.5% sensitivity (14/16). Western blot analysis identified 2 discrepant samples as false positives, suggesting potential ELISA cross-reactivity.

### Virus isolation

Virus isolation attempts were performed using 9 qRT-PCR-positive tick homogenates inoculated onto BHK-21 cell monolayers. While viral RNA was detected in 33% (3/9) of primary cultures (*Ct* range: 31.75-32.36), no observable cytopathic effects developed. The viral RNA load exhibited a progressive decline across successive generations, as evidenced by increasing average *Ct* values (32.25 in P1, 34.88 in P2, and 35.39 in P3). By the fourth generation (P4), viral RNA became undetectable in all samples (*Ct* > 35). These findings indicate that NSDV fails to establish productive infection in BHK-21 cells under current culture conditions.

## Discussion

As an important tick-borne pathogen, NSDV exhibits transmission dynamics that are strongly influenced by climatic conditions. Current epidemiological studies have identified 7 hard tick species as competent vectors, with distinct geographical distributions: *Rhipicephalus appendiculatus*, *R pulchellus*, and *Amblyomma variegatum* serve as the primary vectors in Africa, while *Rhipicephalus haemaphysaloides*, *Haemaphysalis intermedia*, *H wellingtoni*, and *H longicornis* predominate in Asian ecosystems.^13-15^ Notably, recent studies have identified NSDV RNA in ticks parasitizing birds, suggesting their potential role in the virus’s cross-regional dispersal.^16^ Climate change potentiates NSDV transmission through 3 synergistic mechanisms: (1) accelerated tick development and enhanced reproductive capacity under elevated temperatures amplify vector populations^17^^,^^18^; (2) rising temperatures have facilitated the range expansion of tick vectors into previously uninhabited higher latitudes and altitudes^19^; and (3) extended annual tick activity periods from milder winters that increase host exposure frequency.^18^^,^^19^ These drivers collectively facilitate the continuous geographic expansion of NSDV-endemic regions, posing significant emerging threats to global livestock productivity and public health security. In this study, NSDV nucleic acids were detected in tick communities in both Weifang and Yantai of Shandong Province, China, which is the first time that NSDV has been confirmed in the east-central region of Shandong Peninsula. Meanwhile, the prevalence of the virus in the region was further confirmed by serological analysis of domesticated animals near the sampling sites.

Our investigation revealed NSDV circulation in both questing (grassland-collected) and engorged (animal-parasitizing) ticks across Weifang and Yantai, indicating the molecular presence and potential local exposure risk in eastern Shandong’s coastal and central regions. The MIR of NSDV in this study was 1.2%, with no significant difference in tick carrier rates between the 2 sampling sites (*P* > .05). This finding aligns with the NSDV positivity rate (0.87%) reported by Wang et al.^20^ in *H longicornis* ticks from western Shandong. Notably, Shandong Province is an established endemic region for severe fever with thrombocytopenia syndrome virus (SFTSV), and *H longicornis*—the principal vector for both NSDV and SFTSV—is ubiquitously distributed across the province. These observations suggest that NSDV’s actual distribution in Shandong likely surpasses currently reported data, necessitating enhanced surveillance to evaluate its zoonotic threat. While no historical NSDV outbreaks have been documented in Shandong, 2 potential introduction pathways warrant investigation: (1) passive introduction via tick-infested livestock trade, or (2) long-distance dispersal by migratory birds carrying infected ticks. Future studies should prioritize elucidating the dominant transmission mechanism.

Serological analysis revealed NSDV-specific antibodies in sheep from both Weifang and Yantai, Shandong Province, with significantly higher seroprevalence in Weifang (*P* < .05), suggesting this area might represent a potential NSDV circulation area. While previous studies failed to detect NSDV antibodies in Hubei goats,^10^ our study provides the first conclusive evidence of natural NSDV infection in Chinese livestock. These findings indicate established local virus-host maintenance cycles, highlighting potential risks to regional livestock security that warrant enhanced surveillance and preventive measures. To date, no large-scale NSDV outbreaks have been reported in the region, potentially due to subclinical infections in sheep or limited death failing to prompt attention. Notably, we detected NSDV-specific IgG in sheep colostrum, indicating prior exposure of ewes to the virus during pregnancy or pre-lactation. These maternal antibodies are likely transferred to lambs, providing transient protection against early NSDV infection. However, as antibody levels wane, susceptibility increases, underscoring the need for enhanced vector control during this vulnerable period to prevent potential outbreaks. In addition, serological analysis revealed no NSDV-specific antibodies in the tested cattle (*n* = 20), indicating potentially low infection risk in this population; however, the restricted sample size might limit the generalizability of this finding. Nairobi sheep disease virus nucleic acids were undetectable in animal specimens, potentially due to transient viremia or suboptimal sampling timing. To address these limitations, we propose a prospective cohort study of endemic-area livestock (sheep, goats, and cattle), with particular attention to neonates. This longitudinal design will enable monitoring of viral replication dynamics and antibody kinetics under natural exposure conditions, clarifying domesticated animals’ role in NSDV transmission cycles.

Here, we sequenced and characterized a complete NSDV genome obtained through NGS of tick pools. Comparative analysis of the 3 genome segments revealed that the NSDV strain from Weifang City, Shandong Province, China, shares an identical genomic topology and functional organization with other known NSDV strains. Comparative genomic analysis also revealed distinct spatial clustering of NSDV subclades, indicating potential local adaptation. At the same time, these strains from different regions of China showed remarkable similarity in amino acid sequences. In particular, the amino acid sequences of the strains from the western part of the Shandong Peninsula were almost identical to those in this study, suggesting that NSDV strains within a specific geographic range might have originated from a common ancestor or recombined genetically in the same way, and were relatively conserved during evolution. This is also evident in the functional execution of the viral genome, as there is little heterogeneity in the important protein binding sites of conserved structural domains in these viruses from different continents. For example, the polypeptide substrate binding site and active site in the L fragment show little variation. These findings indicate that while NSDV show geographical segregation, they maintain evolutionary conservation in core functional regions, reflecting successful adaptation to local tick vectors with constrained mutation rates at essential protein domains.

Phylogenetic analysis revealed high genetic homology and close evolutionary relationships among Chinese NSDV strains across all 3 genomic segments (L, M, and S), suggesting a low mutation rate and relatively conserved evolution of the virus in China. According to our estimation, NSDV might have undergone low-level macroevolution as an exotic imported virus in China while demonstrating gradual spatial expansion. Notably, the viral strains characterized in this study exhibit high genomic homology with previously reported isolates from Shandong and Jilin, suggesting that they might have originated from a common ancestor. In terms of the global evolutionary, the Chinese strains clustered with the Indian lineage strains in the L and S fragments, while they were distantly related to the Kenyan and Russian strains; however, the evolutionary analysis of the M fragment showed that the Indian lineage strains formed an independent branch, suggesting that the viral membrane proteins underwent a unique evolutionary process locally in India. This phylogenetic divergence illustrates the dual evolutionary dynamics of NSDV: (1) segment-specific evolutionary rates across the viral genome, and (2) geography-dependent adaptation pressures leading to localized niche specialization. In summary, while our findings demonstrate distinct evolutionary patterns among NSDV genomic segments, the complex recombination mechanisms characteristic of Bunyaviruses necessitate additional investigation to fully elucidate their phylogeographic origins and evolutionary trajectories.

Despite repeated attempts, no viable NSDV isolates were obtained in this study. However, this negative result does not preclude the presence of infectious virus, as potential limitations include the unsuitability of the cell culture systems used for NSDV propagation or low viral load in the tick specimens. The failure to obtain viable NSDV isolates suggests that the detected RNA might represent non-infectious fragments or low-titer particles. However, the detection of specific antibodies in local sheep, including colostrum, provides compelling evidence of natural host exposure. While our data strongly points to the presence of NSDV in local tick-ruminant systems, we acknowledge that future successful isolation is required to definitively confirm a sustained transmission cycle.

## Conclusion

This study provides the first confirmed detection of NSDV in ticks from a previously unrecognized epidemic region in China, suggesting wider geographical distribution of the virus than previously recognized. Through comprehensive genomic sequencing and phylogenetic analysis of NSDV strains from *H longicornis* in central and eastern Shandong Peninsula, we characterized the genetic features of circulating strains and identified their relationship to other geographical variants. Most importantly, we report the first evidence of natural NSDV infection in Chinese sheep populations. These findings indicate that NSDV might have established a presence in China, representing a potential threat to livestock industries and public health. Our results highlight the urgent need for enhanced surveillance and control measures targeting sheep and other domestic animals in affected regions.

## Data Availability

The raw metagenomic sequencing data generated in this study have been deposited in the NCBI Sequence Read Archive (SRA) under BioSample accession number SAMN48512720, with individual sequencing runs accessible via SRA accession numbers SRR33580209, SRR33623062, and SRR33623063. The complete genome sequences of NSDV strains identified in this study, including the L (PQ684032), M (PQ684033), and S (PQ684034) segments, along with partial S-segment sequences (PV520060-PV520063), have been submitted to GenBank. These datasets are publicly accessible through the provided accession numbers for academic use and further analysis.

## Off-label antimicrobial declaration

The authors declare no off-label use of antimicrobials.

## Institutional animal care and use committee or other approval declaration

This study was conducted in accordance with the ethical guidelines approved by the Experimental Animal Ethics Committee of Shandong Second Medical University (Approval No. 2021SDL498). The research protocol, designed to investigate NSDV infection in domestic ruminants across Yantai and Weifang regions, received additional approval from local animal husbandry and veterinary administration departments. All procedures strictly adhered to animal welfare principles, with veterinary-supervised standardized protocols implemented to minimize discomfort and prevent unintended death. The study design complies with international standards for ethical animal research and relevant national regulations governing biomedical experimentation.

## Abbreviations

COI: cytochrome oxidase I
FITC: fluorescein isothiocyanate
GP: glycoprotein
IFA: immunofluorescence assay
IPTG: isopropyl β-D-1-thiogalactopyranoside
MIR: minimum infection rate
NGS: next-generation sequencing
NP: nucleoprotein
NSD: Nairobi sheep disease
NSDV: Nairobi sheep disease virus
OD: optical density
ORF: open reading frame
PBS: phosphate-buffered saline
qRT-PCR: quantitative reverse transcription PCR
RdRp: RNA-dependent RNA polymerase
RNA: ribonucleic acid
SDS-PAGE: sodium dodecyl sulfate polyacrylamide gel electrophoresis
SFTSV: severe fever with thrombocytopenia syndrome virus
